# Performance of Antenatal Diagnostic Criteria of Twin-Anemia-Polycythemia Sequence

**DOI:** 10.3390/jcm9092754

**Published:** 2020-08-26

**Authors:** Becky Liu, Erkan Kalafat, Amar Bhide, Basky Thilaganathan, Asma Khalil

**Affiliations:** 1 Fetal Medicine Unit, St George’s University Hospitals, Blackshaw Road, London SW17 0QT, UK; bexliu@doctors.org.uk (B.L.); mail@erkankalafat.com (E.K.); abhide@sgul.ac.uk (A.B.); basky@pobox.com (B.T.); 2 Twins Trust Centre for Research and Clinical Excellence, St George’s University Hospitals, Blackshaw Road, London SW17 0QT, UK; 3 Department of Statistics, Faculty of Arts and Sciences, Middle East Technical University, 06800 Ankara, Turkey; 4 Department of Obstetrics and Gynecology, Faculty of Medicine, Ankara University, 06230 Ankara, Turkey; 5 Vascular Biology Research Centre, Molecular and Clinical Sciences Research Institute, St George’s University of London, Cranmer Terrace, London SW17 0RE, UK

**Keywords:** twin anemia polycythemia sequence, diagnostic criteria, perinatal outcomes, disease progression, antenatal intervention

## Abstract

This study aims to elicit the validation performance of different diagnostic criteria and to evaluate the disease course and perinatal outcomes of pregnancies complicated by twin anemia polycythemia sequence (TAPS). Monochorionic diamniotic (MCDA) twin pregnancies who received serial middle cerebral artery (MCA) peak systolic velocity (PSV) measurements without non-TAPS-related demise or major anomalies were included. Course of disease, antenatal intervention, additional ultrasound features, and perinatal outcomes were compared between each criteria and onset. Forty-nine cases of TAPS and 203 non-TAPS controls were identified. The incidence of TAPS was 19.2%, 15.7%, 7.8%, and 6.3% for ΔPSV MoM > 0.373, ΔPSV MoM > 0.5, traditional, and Delphi consensus criteria, respectively (*p* < 0.001). The incidence of antenatal intervention was 55.1, 62.5, 75.0, and 87.5%, respectively. Furthermore, cases detected according to the Delphi consensus criteria had a higher rate of progression or intervention compared to cases detected with ΔPSV MoM > 0.373 (87.0 vs. 59.0%, *p* = 0.037). TAPS had a significantly higher birth weight discordance than uncomplicated MCDA twins (25.3 vs. 7.3%, *p* < 0.001). Application of four different diagnostic criteria for TAPS leads to significant differences in the incidence, severity, and antenatal intervention. The Delphi criteria identified more severe cases likely to require intervention, and the delta PSV > 0.373 criteria identified milder cases, without a significant impact on neonatal outcomes.

## 1. Introduction

Twin anemia polycythemia sequence (TAPS) is an uncommon complication of monochorionic diamniotic (MCDA) twin pregnancies, due to the presence of miniscule arteriovenous (AV) vascular anastomoses [1]. It is characterized by a discrepancy in hemoglobin levels between the twins without a volume disparity, and can occur spontaneously, in conjunction with twin-to-twin transfusion syndrome (TTTS) or post laser therapy for TTTS [1,2]. It has a reported incidence of spontaneous onset in 3–5% of MCDA twins, and 2–16% in cases after laser photocoagulation for TTTS [2,3,4,5,6]. It is known to be associated with increased perinatal morbidity and mortality [1], and neurodevelopmental impairment [7,8]. The optimal management of TAPS remains uncertain, but strategies varying from fetoscopic laser therapy, intrauterine transfusion with or without partial exchange transfusion, selective reduction, early delivery, to expectant management have been described [7,8]. The available literature is limited by heterogeneity in monitoring protocols, the diagnostic criteria used, and small cohort sizes.

The International Society of Ultrasound in Obstetrics and Gynecology (ISUOG) recommended routine ultrasound monitoring in all MCDA twin pregnancies from 20 weeks gestation, using a diagnostic threshold of a middle cerebral artery peak systolic velocity (MCA PSV) value of >1.5 MoM in the donor, and <1.0 MoM in the recipient [9]. Variations of clinical practice are reflected in other national guidelines, where the National Institute for Health and Care Excellence (NICE) [10] and the Royal College of Obstetricians and Gynaecologists (RCOG) have recommended only performing MCA PSV in complicated MCDA twin pregnancies [11], and the Society for Maternal-Fetal Medicine (SMFM) have not recommended monitoring for TAPS due to lack of robust evidence for improved perinatal outcomes [12]. The correlation between antenatal and postnatal diagnostic criteria is poorly understood. The prognostic implications of antenatal TAPS are unclear but need to be established in order to select those women and babies that would benefit from antenatal intervention.

Recent evidence has questioned the diagnostic accuracy of the traditional diagnostic criteria of >1.5 and <1.0 MoM in the MCA PSV, as recipient twins with PSV >1.0 MoM have been found to be polycythemic at birth, and the delta PSV were felt to be a stronger predictor of hemoglobin discordance at birth [13]. Tollenaar et al. found that an MCA PSV delta of >0.5 MoM had a higher sensitivity and negative predictive value in diagnosing TAPS antenatally than the traditional cut-off of >1.5 and <1.0 MoM [14]. Moreover, Tavares De Sousa et al. reported that a delta of >0.373 MoM was in fact the most optimal predictor of twin Hb discordance of >90th centile at birth [15]. A recent consensus using the Delphi procedure has focused on alleviating heterogeneity and achieving a uniform diagnostic criteria for TAPS. This has proposed that a cut-off of >1.5 MoM in the donor and <0.8 MoM in the recipient, or a PSV delta of >1.0 MoM, is most appropriate for the antenatal diagnosis of TAPS [16].

Our study aims to assess the influence of application of four different diagnostic criteria of TAPS on the disease course and perinatal outcomes in monochorionic twin pregnancies.

## 2. Experimental Section

This is a cohort study of all the monochorionic twin pregnancies presenting to the Fetal Medicine Unit at St George’s Hospital for ultrasound assessment. Patients were identified through a retrospective search through our ultrasound database (ViewPoint version 5.6.26.148, ViewPoint Bildverarbeitung GMBH, Wessling, Germany) from September 2004 to September 2019. Monochorionicity was confirmed at the 11–14 week scan by visualization of the *T*-sign by ultrasound, and the gestational age calculated using the crown-rump length (CRL) of the larger twin, or according to the time of in vitro fertilization (IVF) in women who underwent assisted reproductive techniques [9]. Uncomplicated pregnancies were followed up by ultrasound twice weekly from 16 weeks, and complicated pregnancies underwent more frequent surveillance. Patients who were discharged back to the referring hospital before the third trimester without a diagnosis of TAPS, or those who suffered a single or double pregnancy loss without a prior diagnosis of TAPS were excluded. We also excluded patients with higher order pregnancies, major fetal anomalies not as a consequence of TTTS, those who did not have serial MCA PSV Dopplers measured for both twins, and ones who were lost to follow-up.

All MCA PSV measurements were converted into MoM values using an MCA peak systolic velocity calculator (http://perinatology.com/calculators/MCA.htm). TAPS was diagnosed when two consecutive MCA PSV MoM measurements were in keeping with the following diagnostic criteria: >1.5 MoM in the donor and <1.0 MoM in the recipient (traditional criteria), a delta of >0.5 MoM or >0.373 MoM, or >1.5 MoM in the donor and <0.8 MoM in the recipient or a delta of >1.0 MoM (Delphi criteria). Those who did not fulfil any of these criteria, who underwent serial MCA PSV measurements, were classified as non-TAPS, which consisted of both uncomplicated and complicated pregnancies (e.g., TTTS, selective fetal growth restriction), and were placed into the control group. TTTS was classified according to the Quintero staging system [17], and those with stage II or above underwent fetoscopic laser photocoagulation below 26 weeks, or amniodrainage or early delivery if above 26 weeks. Those with stage I TTTS were either managed conservatively with regular follow-up scans (weekly or more frequent) or underwent intervention if there was evidence of cervical shortening, maternal discomfort, or disease progression [9]. Those with selective fetal growth restriction (sFGR) were classified according to the Gratacós staging system [18] and were followed up with ultrasound surveillance at least every 2 weeks, or weekly in the presence of Doppler abnormalities. Interventions such as fetoscopic laser photocoagulation or selective termination were considered if there was a high risk of fetal demise in the growth restricted twin, in order to reduce the risk of demise or neurological sequelae in the co-twin [9].

Two consecutive positive measurements were required for diagnosis within 1 or 2 weeks, as the false positive rate for detecting fetal anemia using MCA PSV has been reported to be 12% in the second and early third trimesters, and as high as 42% after 34 weeks [19,20]. The onset was classified as spontaneous, post laser for TTTS, or coinciding with the onset of TTTS. Those cases that fulfilled the traditional criteria of >1.5 MoM and <1.0 MoM were staged at the time of onset according to the staging system proposed by Slaghekke et al. [1]. Progression was classified as an increase in MCA PSV MoM in the donor and decrease in the recipient, cardiac compromise of donor, defined as critically abnormal flow (absent or reversed end-diastolic flow in the umbilical artery (AREDF), pulsatile flow in the umbilical vein, increased PI or absent/reversed a-wave in DV), development of fetal hydrops, or intrauterine demise [1]. Regression was determined by an improvement in the MoM values or Doppler indices, or the resolution of hydrops. Natural history was not applicable (NA) when they underwent immediate treatment such as laser on the day of referral and diagnosis. Antenatal interventions for treatment of TAPS took place in the form of laser, intrauterine or partial exchange transfusion, early delivery, or selective reduction, depending on the severity, gestation, previous pregnancy interventions, and patient choice. Diagnosis of TAPS (using any criteria) was not enough for the decision for intervention, but evidence of progression of the abnormal finding on serial assessment was required. The presence or absence of improvement following treatment for those who underwent interventions was documented (classified similarly to regression and progression). All pregnancies classified as TAPS were grouped into the four different diagnostic criteria depending on the MoM values and the criteria met at diagnosis. Additional ultrasound features such as starry sky liver in the recipient (increased brightness of portal venule walls and reduced liver parenchyma echogenicity), placental dichotomy (thick hypoechoic placenta in the donor, and thin placenta with increased echogenicity in the recipient), and cardiomegaly in the donor [9] were noted for each of the TAPS cases according to diagnostic criteria.

Intrauterine demise (IUD) was defined as the death of one or both twins after 20 weeks. Neonatal death (NND) was defined as the death of a newborn up to 28 days of life. Serious neonatal morbidity and mortality included respiratory distress syndrome, blood or exchange transfusion, sepsis, intraventricular hemorrhage, necrotizing enterocolitis, periventricular leukomalacia, or neonatal death. Maternity and neonatal records were reviewed to ascertain the pregnancy and neonatal outcomes data, including gestational age at delivery, birthweight, admission to neonatal unit (NNU), and neonatal morbidity. The birthweight percentiles were computed using the twin chorionicity specific standards reported by Ananth et al. [21].

In order to determine the diagnostic criteria that are most useful in clinical practice, we aimed to compare the criteria that identified the most fetuses at risk of adverse outcome, such as disease progression, presence of additional ultrasound features, need for antenatal intervention, and perinatal mortality.

### Statistical Analysis

Continuous variables were presented as median with interquartile ranges, whereas binary variables were presented as numbers with percentages. Distribution assumptions of the variables were tested with a Shapiro–Wilk test. Group comparisons were made using the *t*-test when normal distribution assumptions were satisfied; otherwise, a Wilcoxon rank sum test was used. The chi-squared test was used for comparison of binary outcomes for unpaired and McNemar’s chi-squared test was used for paired data. *P* values below 0.05 were considered statistically significant. All group comparisons were made with two-tailed tests. The analysis was performed using the R software (R for Statistical Computing (Version 4.0.2)) for statistical computing.

## 3. Results

### Our Study Findings

**Figure 1:**
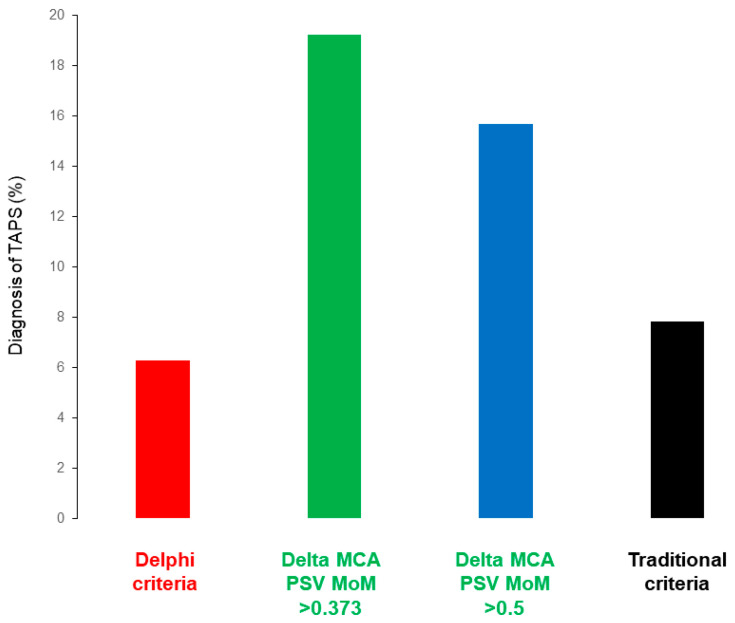
The antenatal incidence of twin anemia polycythemia sequence (TAPS) according to various diagnostic criteria. Delta peak systolic velocity (PSV) multiple of median (MoM)-based criteria increase the number of TAPS diagnoses compared to traditional criteria (Donor twin PSV MoM > 1.5 and Recipient twin PSV MoM < 1.0) and the newly proposed Delphi consensus criteria (*p* < 0.001 for all). There were no significant differences between the traditional criteria and Delphi consensus criteria (*p* = 0.288, McNemar’s chi-squared).

We included 203 MCDA twin pregnancies without an antenatal diagnosis of TAPS and 49 MCDA pregnancies with antenatal diagnosis of TAPS (any criteria). Criteria based on MCA PSV delta MoM increased the incidence of TAPS compared to the traditional criteria (Donor PSV MoM > 1.5 and recipient PSV MoM < 1.0) and Delphi consensus criteria (*p* < 0.001 for all). The incidence of antenatal TAPS varied according to diagnostic criteria. The incidence of TAPS was 19.2%, 15.7%, 7.8%, and 6.3% for ΔPSV MoM > 0.373, ΔPSV MoM > 0.5, traditional, and Delphi consensus criteria, respectively (Figure 1). Of the 16 cases that satisfied the Delphi consensus criteria, 6 met the >1.5 and <0.8 MoM threshold values, 3 were diagnosed using the delta >1.0 MoM cut off, and 7 met both the threshold MoM and delta criteria.

**Figure 2:**
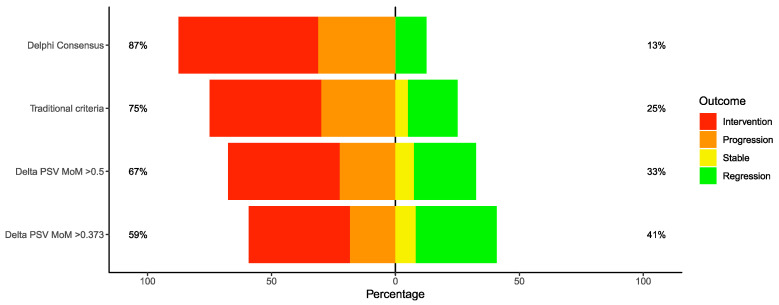
The outcomes of antenatal twin anemia polycythemia sequence (TAPS) cases according to various criteria. Cases diagnosed with Delphi consensus criteria had a higher incidence (87.0% vs. 59.0%) of progression or intervention compared to delta peak systolic velocity multiple of median > 0.373 group (*p* = 0.037).

The baseline characteristics and natural history of the different diagnostic criteria are represented in Table 1. The incidence of spontaneous TAPS was slightly increased with ΔPSV MoM > 0.373 criteria compared to others (46.9% vs. 42.5%, 40.0%, and 37.5%, ΔPSV MoM > 0.5, traditional and Delphi criteria, respectively). The management of TAPS cases was also similar between the groups, but cases detected according to Delphi consensus criteria had an increased rate of intervention (laser photocoagulation, intrauterine transfusion, or selective termination). Intervention was performed on the basis of fetal compromise such as abnormal Dopplers (AREDF, abnormal DV), cardiomegaly, disease progression, fetal hydrops, or other indications such as severe polyhydramnios or cervical shortening if coinciding with TTTS. The incidence of non-expectant management was 87.5%, 75.0%, 62.5%, and 55.1% for Delphi consensus, traditional, ΔPSV MoM > 0.5, and ΔPSV MoM > 0.373 criteria, respectively. Furthermore, cases detected according to Delphi consensus criteria had a higher rate of progression or intervention compared to cases detected with ΔPSV MoM > 0.373 (87.0% vs. 59.0%, *p* = 0.037). The rate of disease regression was also lowest in the Delphi criteria diagnosed cases (13%, 25%, 33%, and 41%, in the Delphi, traditional, ΔPSV MoM > 0.5, and ΔPSV MoM > 0.373, respectively). The rate of intervention, progression, stabilization, or regression according to various diagnostic criteria can be found in Figure 2. Antenatal TAPS cases had a birth weight discordance of 17.4% or more on average depending on the diagnostic criteria. The double livebirth rates were 75.0%, 88.8%, 77.5%, and 81.6% for Delphi consensus, traditional, ΔPSV MoM > 0.5, and ΔPSV MoM > 0.373 criteria, respectively. Neonatal unit admission (range: 70.0% to 75.0%) and serious neonatal morbidity or mortality (range: 35.0% to 43.7%) were similar among the diagnostic criteria despite higher rates of intervention and progression in the Delphi consensus group (Table 1).

Table 2 shows the characteristics, natural history, interventions, and disease course post intervention for spontaneous, post-laser, and TAPS which coincided with TTTS. Spontaneous TAPS were diagnosed slightly later than post-laser and coincided TAPS (25.0, 23.3, and 20.4 weeks, respectively). Expectant management was the preferred choice of treatment for spontaneous and post-laser TAPS (56.5% and 53.3%), whilst almost all patients with coincided TAPS underwent laser for TTTS (9.1% expectantly managed). Disease improvement following intervention, however, was 100% in the TAPS group coinciding with TTTS, whereas these rates were lower for the spontaneous and post-laser groups (80.0% and 42.9%, respectively).

Eighteen twin pairs underwent postnatal hemoglobin examination, as the remaining twins did not have an antenatal diagnosis of TAPS, were not admitted to the neonatal unit, or were lost to follow-up. Table 3 outlines the diagnostic accuracy, sensitivity, and specificity for the traditional, Delphi, and ΔPSV MoM > 0.5 groups, but as all 49 cases met ΔPSV MoM > 0.373 criteria, diagnostic accuracy could not be calculated in this group. Reticulocyte count ratio was not performed as this is not a part of our routine full blood count analysis. There were no significant differences in the postnatal hemoglobin differences between fetuses who met the Delphi Criteria by MoM limit criteria or by Delta MoM value (*P* = 0.636). The odds of having a postnatal hemoglobin difference greater than 8 mg/dL were higher in the expectant management group compared to intervention group but the difference did not reach statistical significance (OR: 5.0, 95% CI: 0.57–112.0). The insignificant difference is likely a reflection of low numbers included in the analysis.

**Figure 3:**
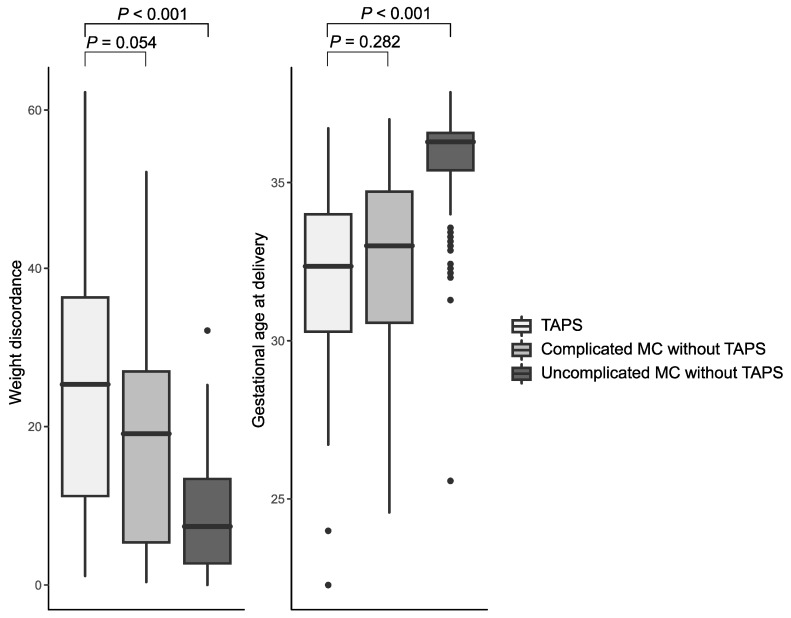
Box plots of gestational age at delivery (weeks) and birth weight discordance (%) in monochorionic diamniotic (MCDA) twins without twin anemia polycythemia sequence (TAPS) and twins with antenatal diagnosis of TAPS (any criteria). The MCDA twins without TAPS were stratified according to whether they were complicated otherwise (selective fetal growth restriction, twin–twin transfusion syndrome). Fetuses with antenatal diagnosis of TAPS delivered earlier (median: 32.4 vs. 33.0 and 36.3 weeks, TAPS vs. complicated and uncomplicated MCDA, *p* = 0.282 and *p* < 0.001, respectively) and had a higher birth weight discordance at birth (25.3% vs. 19.1% and 7.3%, TAPS vs. complicated and uncomplicated MCDA, *p* = 0.054 and *p* < 0.001, respectively).

Cases with antenatal diagnosis of TAPS were delivered earlier and had higher birth weight discordance compared to a control group of MCDA pregnancies without antenatal TAPS (Figure 3). The MCDA twins without TAPS were stratified according to whether they were complicated otherwise (selective fetal growth restriction, twin-to-twin transfusion syndrome etc.), or uncomplicated. Fetuses with antenatal diagnosis of TAPS delivered earlier (median: 32.4 vs. 33.0 and 36.3 weeks, TAPS vs. complicated and uncomplicated MCDA, *p* = 0.282 and *p* < 0.001, respectively) and had a higher birth weight discordance at birth (25.3% vs. 19.1% and 7.3%, TAPS vs. complicated and uncomplicated MCDA, *p* = 0.054 and *p* < 0.001, respectively).

**Figure 4:**
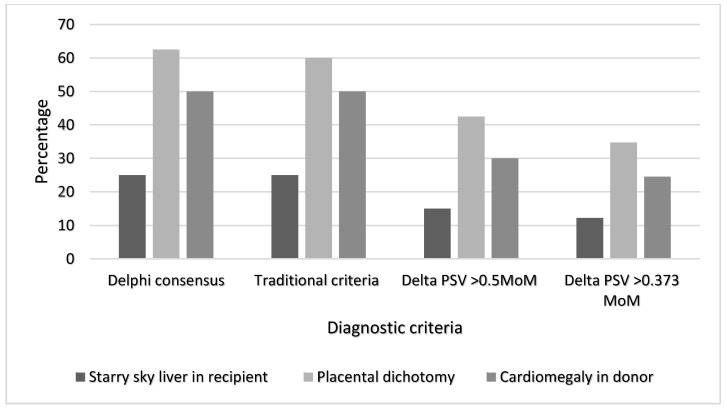
Bar chart showing the percentage presence of additional ultrasound markers such as starry sky liver in the recipient, placental dichotomy, and cardiomegaly in the donor in each of the four diagnostic criteria (Delphi consensus *n* = 16, Traditional criteria *n =* 20, Delta PSV > 0.5 MoM *n =* 40, Delta PSV > 0.373 MoM *n* = 49). These features were similarly prevalent in the Delphi and traditional criteria, but less prevalent in the delta PSV criteria.

Starry sky liver was seen in the recipient twin in 25%, 25%, 15%, and 12.2% in the Delphi, traditional, ΔPSV MoM > 0.5, and ΔPSV MoM > 0.373 criteria groups, respectively. Placental dichotomy was seen in 62.5%, 60%, 42.5%, and 34.7% in these groups, respectively, and cardiomegaly in the donor twin was present in 50%, 50%, 30%, and 24.5% of the Delphi, traditional, ΔPSV MoM > 0.5, and ΔPSV MoM > 0.373 criteria groups, respectively (Figure 4).

## 4. Discussion

### 4.1. Summary of the Study Findings

The incidence of TAPS differed according to the diagnostic criteria used, where the ΔPSV MoM > 0.373 criteria yielded the highest incidence, and the Delphi criteria the lowest. Cases classified as TAPS according to the Delphi consensus criteria had the highest rate of progression and intervention, and were therefore more likely to identify fetuses at risk. The double livebirth, neonatal care unit admission, or serious neonatal morbidity and mortality rates were similar between the different criteria. Antenatally diagnosed TAPS were delivered earlier and had a higher rate of birthweight discordance than the uncomplicated MCDA twins without TAPS. Additional ultrasound features were more prevalent in the Delphi and traditional criteria groups than in the ΔPSV MoM groups.

### 4.2. Interpretation of Study Findings and Comparison with Existing Literature

TAPS was described by Robyr et al. in 2006, where many twins were found to have an MCA PSV above 1.5 MoM in one twin and below 0.8 MoM in the other following laser treatment for TTTS [4]. Following this, it was proposed that the diagnostic criteria be corrected to >1.5 MoM and <1.0 MoM in the donor and recipient, respectively, as it was noted that some recipients still required partial exchange transfusions after delivery, or suffered intrauterine demise despite a stable MCA PSV at 1.0 MoM [1]. Recent studies have found that this criterion yielded a sensitivity of 46% and specificity of 100%, with 100% and 70% positive (PPV) and negative predictive values (NPV), respectively. By incorporating the diagnostic criteria of ΔPSV > 0.5 MoM, however, the sensitivity and NPV were increased to 83% and 88%, respectively [14]. Similarly, the ΔPSV >0.373 MoM criteria had an increased sensitivity of 93.3%, a specificity of 95.7%, and a 70% and 99.3% PPV and NPV [15]. The Delphi consensus thereby incorporated criteria that consisted of both a PSV MoM cut-off, as well as discordance, due to the findings that discordance may be of more significance than cut-off values alone, but further research is required to validate the optimum discordance used for diagnosis [13,16].

Our results have shown that a higher number of cases fulfilled the ΔPSV > 0.373 MoM criteria, but 44.9% were managed expectantly, with 41% that remained stable or spontaneously regressed. Conversely, far fewer cases fulfilled the Delphi consensus criteria, but these cases appeared more severe, as 87% progressed or required intervention, compared to 59% from the ΔPSV > 0.373 MoM group (*p* = 0.037). Therefore, the implementation of the ΔPSV > 0.373 MoM criteria may lead to overdiagnosis and potentially unnecessary intervention, whereas the Delphi criteria has a potential risk of under or late diagnosis. The rate of double live births, however, has not shown a significant difference in any of the groups.

Stagnati et al. demonstrated that MCA PSV MoM discrepancy can be an independent predictor of selective fetal growth restriction (sFGR) and birthweight (BW) discordance of >25%. They found that a 0.30 MoM discrepancy in MCA PSV, particularly in the third trimester, provided the optimal cut-off, with a 70% and 69% sensitivity and specificity for sFGR, and 83% and 72% sensitivity and specificity for BW discordance >25% [22]. Furthermore, a 0.1 MoM increase in PSV discrepancy was found to have a 21-fold higher risk of sFGR. Our results support the findings by Stagnati et al., where we have demonstrated that pregnancies with an antenatal diagnosis of TAPS (any criteria) had a significantly higher birth weight discordance at birth compared to uncomplicated MCDA pregnancies, and a higher birth weight discordance than complicated MC pregnancies, although not statistically significant.

Tollenaar et al. studied the presence of additional ultrasound features in MCDA twins with TAPS using the ΔPSV MoM > 0.5 criteria, and found that 66% had features of starry sky liver in the recipient, 44% had placental dichotomy, and 70% had cardiomegaly in the donors [23]. Our data showed a lower presence of starry sky liver and cardiomegaly, and a similar presence of placental dichotomy in the same group of patients. Both the ΔPSV MoM groups had a lower incidence of additional ultrasound features than the Delphi and traditional criteria groups, possibly due to a milder form of the condition, and earlier detection prior to the development of these features.

### 4.3. Clinical and Research Implications

So far, there is limited consensus on the most optimal diagnostic criteria for TAPS, and published data are restricted by the small sample size. Our study presents the first comparison of the newly proposed criteria. The Delphi criteria showed a low sensitivity and high specificity, whilst the delta PSV criteria showed a high sensitivity but low specificity. The diagnostic accuracy should be interpreted with extreme caution as many patients underwent antenatal treatment, particularly in the Delphi consensus group, and those with postnatal hemoglobin performed were limited to babies who were diagnosed with antenatal TAPS or admitted to the neonatal unit, and is, therefore, likely to be unreliable. Further research is required with a larger cohort and postnatal confirmation in order to provide more robust evidence. The natural history of disease progression and need for intervention demonstrated in our study has, however, shown significant differences between the different criteria, suggesting a difference in the disease stage or severity where TAPS would be identified.

The existing literature works have all excluded a diagnosis of TAPS when a polyhydramnios/oligohydramnios sequence was present, therefore, no cases of TAPS coinciding with TTTS have been studied or compared with spontaneous or post-laser TAPS. Our results suggest that this group of TAPS may respond better to laser treatment compared to the other groups, however, due to the small numbers in our study, further research is required to draw more robust conclusions. This differentiation in treatment outcomes with this group of patients may aid in management planning and future patient counselling.

The association between TAPS and BW discordance could be secondary to the fact that FGR babies have been found to have higher PSV values [24] or may be due to unbalanced placental sharing [25]. Unbalanced blood flow through placental anastomoses in TAPS may be slower and more chronic than TTTS, and may allow for fetal compensation in the form of FGR in one twin to occur. However, as the ΔPSV criteria is based on a PSV discordance rather than the presence of anemia and polycythemia, TAPS may be falsely diagnosed in cases of sFGR (reflection of unequal placental sharing rather than true anemia and polycythemia), as reflected in our results that the ΔPSV criteria detected more BW discordance than the Delphi or traditional criteria (Table 1). Nevertheless, it may still be reasonable to monitor for signs of sFGR following a diagnosis of a PSV discordance or TAPS, or to perform serial MCA PSV measurements to monitor for the development of TAPS following a diagnosis of sFGR.

### 4.4. Strengths and Limitations

Our study provides the first comparison of the disease course and perinatal outcomes of the newly proposed delta diagnostic criteria, Delphi consensus criteria, and the traditional diagnostic criteria. As the Delphi criteria were published in the last year, there was minimal possibility of operator bias. Given the rarity of the condition, our study has included a relatively large patient cohort, which is compared with a large control group of unselected MCDA twin pregnancies. However, this sample size remains too small to draw robust conclusions, therefore, multicenter trials are required to conduct further adequate screening studies.

The diagnostic accuracies of the diagnostic criteria are likely subject to bias, due to the fact that many patients underwent antenatal intervention, and those not requiring NNU admission or the mild cases without an antenatal diagnosis of TAPS will not have undergone hemoglobin testing. Furthermore, neonatal outcomes could not be reliably reported, due to the influence of interventional bias, as some severe cases of TAPS may have been treated with laser, resulting in a good recovery, and may have more optimal outcomes than other milder cases which have been managed expectantly. The lack of difference in neonatal outcomes may be due to the confounding effect of prematurity. The presence of additional ultrasound features was recently reported, and as routine reporting of these features has not been recommended by national guidance, the presence of these features was limited by operator bias, as many may not have undergone routine examination. MCA PSV (>1.5 and <1.0 MoM) has been shown to be predictive of severe anemia or polycythemia [26], but the correlation with mild–moderate cases are unclear. In essence, due to the variability in the correlation of MCA PSV with postnatal biochemistry, and the influence of antenatal interventions, methods to reliably show superiority of one criterion over another are likely difficult to achieve.

Most cases of TAPS discharged for continued care in referring units were in keeping with the ΔPSV criteria, but those diagnosed using the traditional or Delphi criteria were also discharged upon normalization of the MCA PSV, or if the condition for referral (TTTS, sFGR) had improved, due to easier access to care. MCA PSV is often not routinely performed in referring units, which limits our access to the disease course following discharge, however, recent national guidance has recommended performance of serial MCA PSV Dopplers following complicated twin pregnancies [10,11]. Diagnosis of TAPS in referring units are mostly in keeping with the traditional criteria, or if MCA PSV was not performed, a diagnosis of cardiomegaly, abnormal DV, or hydrops will also trigger a referral back to our unit. As the cases diagnosed using ΔPSV criteria may have been lost to follow-up following discharge, this may have led to an increased proportion of the more severe cases (Delphi and traditional) in our study population. However, as this study is not focused on screening, but a comparison of outcomes of the different diagnostic criteria, this will unlikely impact our study conclusions.

Patients who were discharged to the referring units for follow-up and delivery may have had better outcomes as they no longer required tertiary level neonatal care, therefore, our existing outcome data may be biased towards a more high risk group, thus, with worse outcomes. However, this group of patients with diagnosed TAPS is compared to a control group of non-TAPS patients who may also have suffered complications such as TTTS and sFGR, requiring delivery in a tertiary level neonatal unit, which also renders them as high risk pregnancies.

## 5. Conclusions

Application of four different diagnostic criteria for TAPS leads to significant differences in the incidence, severity, and antenatal intervention. The Delphi consensus criteria identified the cases of TAPS with the highest progression and intervention rates, and the ΔPSV > 0.373 MoM criteria identified the lowest. Further multicenter studies are required to provide more robust evidence into the diagnostic accuracy and neonatal outcomes of the various diagnostic criteria.

## Figures and Tables

**Table 1:** Characteristics and natural history of antenatal twin anemia polycythemia sequence (TAPS) cases diagnosed according to the different diagnostic criteria.

	Delphi Consensus (*n* = 16)	Traditional Criteria (*n* = 20)	Delta PSV MoM > 0.5 (*n* = 40)	Delta PSV MoM > 0.373 (*n* = 49)
Maternal age in years, median (IQR)	35.0 (26.5–36.2)	34.5 (24.7–36.5)	32.5 (26.0–36.0)	33.0 (27.0–36.0)
Body mass index in Kg/m^2^, median (IQR)	24.9 (22.7–25.5)	24.3 (22.9–25.5)	23.4 (21.7–25.4)	23.7 (21.8–27.1)
Gestational age at diagnosis in weeks, median (IQR)	23.1 (19.6–24.1)	23.4 (19.9–24.4)	23.4 (19.4–25.6)	23.6 (19.7–27.1)
TAPS onset				
Spontaneous	6 (37.5)	8 (40.0)	17 (42.5)	23 (46.9)
Post-laser	6 (37.5)	8 (40.0)	14 (35.0)	15 (30.6)
Coincided with TTTS #	4 (25.0)	4 (20.0)	9 (22.5)	11 (22.5)
Management *				
Expectant	2 (12.5)	5 (25.0)	15 (37.5)	22 (44.9)
Laser	10 (62.5)	10 (50.0)	19 (47.5)	21 (42.9)
Intrauterine transfusion	3 (18.7)	2 (10.0)	4 (10.0)	4 (8.2)
Selective termination	2 (12.5)	4 (20.0)	3 (7.5)	4 (8.2)
Immediate delivery	1 (6.2)	1 (5.0)	2 (5.0)	2 (4.4)
Gestational age at delivery in weeks, median (IQR)	32.4 (28.4–33.9)	32.0 (30.0–34.0)	32.7 (29.3–34.0)	32.4 (30.4–34.0)
Both twins live born	12 (75.0)	16 (88.8)	31 (77.5)	40 (81.6)
Double IUD	1 (6.2)	1 (5.0)	1 (2.5)	1 (2.0)
Missing data	0 (0.0)	2 (10.0)	2 (5.0)	2 (4.1)
Birth weight—larger twin	1610 (1321–1893)	1768 (1354–1952)	1720 (1372–2022)	1702 (1430–2016)
Birth weight centile—larger twin	30.1 (15.1–56.2)	36.1 (23.1–46.9)	36.3 (25.6–48.2)	38.8 (24.9–50.2)
Birth weight—smaller twin	1155 (982–1567)	1240 (995–1596)	1230 (970–1622)	1214 (958–1543)
Birth weight centile—smaller twin	5.9 (0.0–16.8)	5.7 (0.0–16.8)	1.8 (0.0–16.7)	1.5 (0.0–16.3)
Missing birthweight data	4 (25.0)	4 (20.0)	9 (22.5)	9 (18.4)
Weight discordance	17.4 (7.5–28.9)	19.4 (7.5–36.3)	22.6 (10.1–32.1)	25.3 (11.2–36.3)
NNU admission, any baby	11 (73.3)	14 (70.0)	28 (64.1)	36 (75.0)
Serious neonatal morbidity or mortality, any baby **	6 (40.0)	7 (35.0)	17 (43.6)	21 (43.7)

* Few patients had more than one method of management. ** Respiratory distress syndrome, blood or exchange transfusion, sepsis, intraventricular hemorrhage, necrotizing enterocolitis, periventricular leukomalacia, or neonatal death. # Coincided with TTTS indicates that TAPS was diagnosed simultaneously with coexisting TTTS. IQR—interquartile range; TTTS—twin to twin transfusion syndrome; IUD—intrauterine demise; NNU—Neonatal unit.

**Table 2:** Characteristics, natural history, intervention, and disease course following intervention in spontaneous onset TAPS, post-laser, and TAPS coincided with TTTS.

	Spontaneous (*n* = 23)	Post-Laser (*n* = 15)	Coincided with TTTS # (*n* = 11)
Maternal age in years, median (IQR)	32.0 (26.0–36.0)	32.0 (29.5–34.5)	33.0 (30.5–36.0)
Body mass index in Kg/m^2^, median (IQR)	23.9 (22.0–28.7)	22.4 (20.8–24.3)	25.3 (22.8–26.4)
Gestational age at diagnosis in weeks, median (IQR)	25.0 (22.6–29.7)	23.3 (19.6–26.8)	20.4 (18.0–21.9)
Natural history			
Progression	6 (26.1)	3 (20.0)	0 (0.0)
Regression	9 (39.1)	6 (40.0)	1 (9.1)
Stable	2 (8.7)	2 (13.3)	0 (0.0)
NA (immediate treatment)	6 (26.1)	4 (26.7)	10 (90.9)
Management *			
Expectant	13 (56.5)	8 (53.3)	1 (9.1)
Laser	7 (30.4)	4 (26.7)	10 (90.9)
Intrauterine transfusion	1 (4.3)	3 (20.0)	0 (0.0)
Selective termination	2 (8.7)	1 (6.7)	0 (0.0)
Immediate delivery	1 (4.3)	1 (6.7)	0 (0.0)
Disease course following intervention			
Improved	8 (80.0)	3 (42.9)	10 (100.0)
Deteriorated	1 (10.0)	2 (28.6)	0 (0.0)
Stable	0 (0.0)	0 (0.0)	0 (0.0)
Delivery post intervention	1 (10.0)	2 (28.6)	0 (0.0)
Gestational age at delivery in weeks, median (IQR)	32.8 (30.6–34.1)	32.4 (30.2–34.0)	31.7 (29.9–32.4)

* Few patients had more than one treatment. # Coincided with TTTS indicates that TAPS was diagnosed simultaneously with coexisting TTTS. IQR—interquartile range; NA: non-applicable—patients who underwent intervention immediately following time of diagnosis.

**Table 3:** Diagnostic accuracy of traditional, delta PSV > 0.5 MoM, and Delphi consensus criteria, according to postnatal hemoglobin *.

	Accuracy (95% CI)	Sensitivity (95% CI)	Specificity (95% CI)	TP	TN	FP	FN
Traditional criteria	44.4 (21.5–69.2)	14.3 (0.36–57.9)	63.6 (30.8–89.1)	1	7	4	6
Delta PSV > 0.5 MoM	50.0 (26.0–74.0)	85.7 (42.1–99.6)	27.3 (6.0–61.0)	6	3	8	1
Delphi consensus	55.6 (30.8–78.5)	28.6 (3.7–71.0)	72.7 (39.0–94.0)	2	8	3	5

CI—confidence interval; TP—true positive; TN—true negative; FP—false positive; FN—false negative. The numbers in accuracy, sensitivity, and specificity columns are percentages. The numbers in the remainder of columns are count data. * This information is available in only 18 fetuses. Predictive accuracy analysis for delta PSV > 0.373 MoM is not possible because the criteria applies to all 49 fetuses in the study.
